# ELISPOTs Produced by CD8 and CD4 Cells Follow Log Normal Size Distribution Permitting Objective Counting 

**DOI:** 10.3390/cells4010056

**Published:** 2015-01-20

**Authors:** Alexey Y. Karulin, Kinga Karacsony, Wenji Zhang, Oleg S. Targoni, Ioana Moldovan, Marcus Dittrich, Srividya Sundararaman, Paul V. Lehmann

**Affiliations:** 1 Cellular Technology Ltd., 20521 Chagrin Blvd. Shaker Heights, OH 44122, USA; E-Mails: kinga.karacson@immunospot.com (K.K); wenji.zhang@immunospot.com (W.Z.); oleg.targoni@immunospot.com (O.S.T.); ioana.moldovan@immunospot.com (I.M.); srividya.sundararaman@immunospot.com (S.S.); pvl@immunospot.com (P.V.L.).; 2 M.D., Ph.D., Biocenter, University of Wuerzburg, Am Hubland, 97074 Würzburg, Germany; E-Mail: marcus.dittrich@biozentrum.uni-wuerzburg.de

**Keywords:** ELISPOT, Normal Distribution, software, spot size, gating, cytokines, IFN-γ, IL-2, IL-4, IL-5, IL-17, T cells, CD4, CD8

## Abstract

Each positive well in ELISPOT assays contains spots of variable sizes that can range from tens of micrometers up to a millimeter in diameter. Therefore, when it comes to counting these spots the decision on setting the lower and the upper spot size thresholds to discriminate between non-specific background noise, spots produced by individual T cells, and spots formed by T cell clusters is critical. If the spot sizes follow a known statistical distribution, precise predictions on minimal and maximal spot sizes, belonging to a given T cell population, can be made. We studied the size distributional properties of IFN-γ, IL-2, IL-4, IL-5 and IL-17 spots elicited in ELISPOT assays with PBMC from 172 healthy donors, upon stimulation with 32 individual viral peptides representing defined HLA Class I-restricted epitopes for CD8 cells, and with protein antigens of CMV and EBV activating CD4 cells. A total of 334 CD8 and 80 CD4 positive T cell responses were analyzed. In 99.7% of the test cases, spot size distributions followed Log Normal function. These data formally demonstrate that it is possible to establish objective, statistically validated parameters for counting T cell ELISPOTs.

## 1. Introduction

The primary goal of ELISPOT analysis is to accurately establish the frequency of antigen-specific T cells within the total population of peripheral blood mononuclear cells, PBMC [1]. Their frequency measured in the blood, *ex vivo*, reveals the clonal size of antigen-specific T cells *in vivo*, *i.e.* the magnitude of the T cell response to an antigen. Whereas a given blood sample will contain a discrete number of antigen-specific T cells, ensuring precise measurements of such remains challenging. Immunoassay proficiency panels attempting to measure the frequencies of antigen-specific T cells within identical PBMC sample aliquots, at multiple testing facilities, have reported alarming discrepancies [2]. For tetramer assays, results varied by more than 100-fold, intracytoplasmic staining assays (ICS) varied by 20-fold, and ELISPOT assays 35-fold in their measurements of antigen-specific T cell frequencies. This variability resulted from both un-harmonized assay protocols and data analysis. Even when using dedicated ELISPOT counting software, counting parameters established subjectively by different investigators result in high variability of spot counts. If, however, objective automated spot-size gating could be applied to ELISPOT analysis, one could eliminate subjective judgment from the counting process, and such assays would reliably measure antigen-specific T cells with high precision. 

T cell ELISPOT assays, irrespective of the cytokines measured, always result in a wide range of spot sizes. This variability in ELISPOT sizes is a function of the different quantities of cytokine secreted by individual T cells following antigen stimulation, and is invariably seen with intracytoplasmic staining as well [3,4]. In addition to the cognate spots generated by the antigen-specific T cells, some spots can be produced by clusters of T cells, others by bystander cells (like NK cells in IFN-γ assays) and some might represent assay artifacts. Similar to flow cytometry, in order to ensure the accuracy of T cell frequency measurements, it is necessary to set upper and lower spot size thresholds (gates) for ELISPOT counting, to distinguish spots produced by individual antigen-specific T cells from clusters of such cells (upper gate), as well as from non-specific background spots (lower gate). If spots produced by individual T cells follow a specific (known) theoretical distribution function, upper and lower gates can be automatically calculated by ELISPOT counting software based on objective statistical criteria. 

We set out to establish the basic criterion for accurate ELISPOT data analysis by experimentally investigating the scientific principles underlying these assays. By seeding T cell clones on a monolayer of antigen presenting cells (APC), we were able to examine the cytokine secretion signature of defined numbers of individual T cells in ELISPOT assays [5]. ELISPOTs generated by cloned T cells covered a wide range of sizes. However, the size distribution of these spots showed the symmetric bell-shaped curve, in logarithmic scale, characteristic of Gaussian (Normal) distribution. The average spot sizes as well as the ranges of these experimental distributions varied depending on the dose of antigen used and the length of time since the previous stimulation. Yet in all cases, the observed spot sizes closely followed Log Normal distribution [5]. 

*Ex vivo* T cell responses are rarely clonal. Therefore, we set out to observe spot size distributions for real T cell antigen-recall responses in humans and mice. All such data analyzed so far showed the bell-shaped distribution of spot sizes. For human CD8 T cells, this distribution was observed for individual EBV, HCMV, HIV, influenza virus peptides, as well as peptide pools [6,7,8,9,10,11]. For human CD4 T cells, the distribution was seen for Candida, HCMV, Mumps, and Vaccinia virus antigens [6,7,8,12,13,14], and it was also seen for OVA and MOG in Balb/c and B6 mice [10].

Despite the bell-shaped curves, and their Log Normal distribution, observed in the above mentioned literature, there was no statistical assessment *i.e.*, “goodness of fit” test that was formally performed. Also, the above referenced studies were conducted on relatively few test subjects (generally less than 10). Given the importance for definitively establishing whether T derived ELISPOTs follow Log Normal distribution (and therefore whether they can be counted objectively), we set out to conduct a systematic survey from a substantial library of ELISPOT data communicated in this paper. 

We tested 172 healthy human donors for recall responses induced by defined peptide and protein antigens. A total of 334 positive ELISPOT responses generated by CD8 cells, and 80 individual CD4 recall responses have been identified. The responses were selected based of high numbers of antigen-induced spots (>1000 spots cumulatively in up to 20 replicate wells) to permit stringent statistical analysis of spot size distributions. Using the Kolmogorov–Smirnov test we observed that the donors’ responses to antigen stimulation displayed Log Normal size distributions. Log Normal distributions allow objective size gating based on statistical principles ensuring accurate spot counts. 

## 2. Experimental Section 

### 2.1. PBMC 

Cryopreserved human PBMC of 172 healthy donors were obtained from a library of PBMC (ePBMC, CTL CP1) offered by Cellular Technologies Ltd. (CTL, Shaker Heights, OH, USA). In addition to high-resolution HLA-typing, these PBMC had been previously characterized for T cell reactivity to a panel of antigens. Prior to testing, the PBMC cryovials, stored in liquid N_2_ vapor phase, were transferred to dry ice in Styrofoam containers for transport to, and short term storage in, the testing laboratory. The cells were thawed following a protocol that we have established to provide the optimal recovery and functionality for cryopreserved PBMC [15]. Specifically, to rapidly warm up to 37 °C, the cryovials were placed for 8 min into a 37 °C glass bead bath (CTL-BB-001). The cryovials were inverted twice to re-suspend the cells, and the 1 mL cell suspension contained in each cryovial (10 × 10^6 ^cells) was gently aspirated utilizing a wide-bore 2 mL pipette and transferred into a 15 mL V bottom Falcon tube. To recover the residual cells, the cryovials were rinsed by adding 1 mL 37 °C warm CTL Anti-Aggregate Wash™ Medium (CTL-AA-005) containing benzonase. An additional 8 mL CTL Anti-Aggregate Wash™ Medium at 37 °C was added to the 15 mL tube at a rate of 2 mL per 5 s. After washing, a sample of the cells was counted while also determining viability using the Live/Dead/Apoptotic cell counting platform by CTL (Cat# CTL-LDAC-100). PBMC were washed twice in 10 mL CTL-Test™ Medium (CTLT-005) and re-suspended at a final concentration of 3 × 10^6^ PBMC/mL in the same medium. The freshly thawed PBMC were plated into an ELISPOT assay plate within 1 h of thawing. 

### 2.2. ELISPOT Assays

Human Interferon-γ ImmunoSpot® kits (CTL-HIFNG-1/5M), human IL-2- (CTL-HIL21M/5), IL-4- (CTL-HIL4-1M/5), IL-5- (CTL-HIL5-1M/5), and IL-17 kits (CTL-HIL17-1M/5) were obtained from CTL. These cytokine assays were each performed according to the manufacturer’s recommendations. The individual peptide antigens from the CEF32 pool (CTL Cat # CEF32-07-001-32) were plated at 4 μg/mL, whereas EBV and CMV Grade 2 antigens (Microbix Biosystem Inc. Ontario, Canada) were plated at 50 μg/mL. The antigens were dissolved in either CTL-Test™ Medium (CTLT-005) or signal-enhancing CTL-Test Plus™ Medium (CTLTP-005) as specified. The antigens were plated first, before the PBMC, into the capture antibody-coated assay plate in a volume of 100 µL per well. The plates containing the antigens and medium control were stored at 37 °C in a CO_2_ incubator until the cells were ready for plating. The thawed PBMC, adjusted in CTL Test Medium to 3 × 10^6^ PBMC/mL were plated at 100 µL/well using wide-bore pipette tips. Up to 20 replicate wells were used for individual donor/antigen combinations to accumulate at least 1000 spot for a single spot-size distribution. Plates were gently tapped on each side to ensure even distribution of the cells as they settled and were incubated at 37 °C in a CO_2_ incubator. The incubation period for IFN-γ and IL-2 assays was 24 h, for IL-4 and IL-5 it was 48 h, and for the IL-17 assay it was 72 h. Subsequently, the plates were washed, and the detection reagents were added. Following completion of the ELISPOT protocol, the plates were air dried in a laminar flow hood prior to analysis.

ELISPOT plates were scanned using an ImmunoSpot® S6 Ultimate Analyzer by CTL and analyzed with ImmunoSpot® v.5.3 Software. Spots were counted in Basic Count™ mode using the local dynamic background correction and “Normal Object Type”. The spot size distributions were built from cumulative counts of groups of replicate wells to obtain the specified sample size. The “Sensitivity” was set to detect spots exceeding background well staining. All detected spots were analyzed without size gating. 

The ImmunoSpot® Software automatically translates spot sizes from pixels into millimeters, making spot size measurements independent of the image pixel resolution. The accuracy of spot size measurement, however, is proportional to the image resolution. Spot size data analysis was performed using standard ImmunoSpot® system resolution of 512 × 512 pixels which is precise enough to detect spots about 30 microns in diameter. The ImmunoSpot® system is capable of up to 7000 × 7000 pixel resolution, which however does not add to the precision of ELISPOT analysis. 

### 2.3. Statistical Evaluation

To analyze experimental spot size distributions, the Kolmogorov–Smirnov goodness of fit test was used. The Kolmogorov–Smirnov null hypothesis states that samples come from a normally distributed population. Resulting p-values equal to or higher than the significance level α = 0.05 indicate acceptance of the null hypothesis. Mean and standard deviation for experimental size distributions cannot be evaluated in the Kolmogorov–Smirnov goodness of fit test directly, doing so may lead to “overoptimistic” test results. Means and standard deviations were calculated independently using 1000–3000 spots for maximal precision; the number of replicate wells used depended on the spot counts per well and was between 3–20. Of these, three hundred spots were randomly selected and subjected to the Kolmogorov–Smirnov goodness of fit test. Three hundred spots is optimal for statistical evaluation using the Kolmogorov–Smirnov goodness of fit test for normality: much lower counts do not provide enough information to build good spot-size histograms, whereas much higher numbers result in a low power (sensitivity) of this test do detect normality. With much larger sample sizes even minor random deviations from Normal distribution will result in low p-values. The statistical analysis was performed using the XLSTAT software suite.

## 3. Results and Discussion

### 3.1. T cell ELISPOT Assays Produce a Wide Range of Spot Sizes that Follow Bell Shaped Size Distributions

To analyze spot size distributions in T cell ELISPOT assays, we studied recall responses in 172 healthy human donors. The donors were tested for the individual peptides that constitute the CEF pool, *i.e.*, 32 well defined MHC-class I restricted determinants recognized by CD8 cells. A total of 334 moderate to strong positive donor/peptide combinations were detected that cumulatively provided at least 1000 spots each. The PBMC of the 172 donors were also tested for protein antigen-induced recall responses to EBV- and HCMV- grade 2 antigen, the latter representing UV-inactivated virions, *i.e.* lacking infectivity. In these cases, the viral proteins are processed as extracellular antigens, and are presented on HLA-class II molecules to CD4 cells. By depleting CD4/CD8 cells from PBMC we showed that protein antigen-induced cytokine responses result from activated CD4 cells (data not shown), as was expected from established principles of antigen presentation. In addition to IFN-γ, IL-2, IL-4, IL-5 and IL-17 were measured for analysis of the cytokine signatures of Th1, Th2 and Th17 cells. A total of 80 positive antigen/donor combinations were identified, which yielded moderate to strong CD4 responses that provided sufficient spots for stringent statistical analysis. 

**Figure 1:**
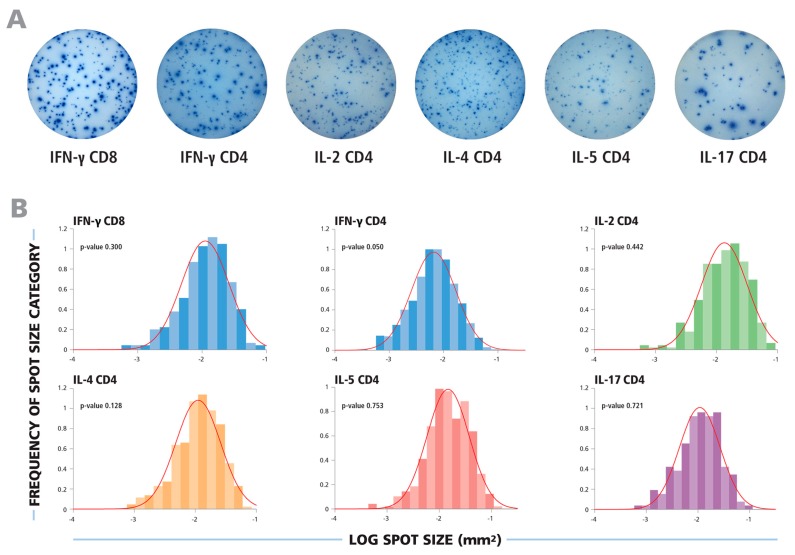
The spot size distributions for different cytokines follow Log Normal function. (**A**) Representative images of ELISPOT wells are shown for cytokines produced by CD8 or CD4 cells, as specified. (**B**) The experimental size distributions of typical recall responses are shown as histograms for the specified cytokines with the theoretical Normal functions overlaid as red lines. To test for the normality of the spot size distributions, the Kolmogorov–Smirnov goodness of fit test was used.

Typical ELISPOT wells for the cytokines tested are shown in Figure 1A. In each case, a wide range of spot sizes was observed, spanning 100 to 300-fold difference in spot size areas. Using subjective criteria to determine the minimum and maximum spot sizes, effectively eliminating bystander responses and estimating spots resulting from T cell clusters, will inevitably lead to highly variable spot counts and poor count reproducibility between different investigators and laboratories. We therefore systematically tested whether spot sizes followed a defined theoretical distribution function that would permit statistics-based gating for the smallest and largest spots produced by individual T cells. 

In Figure 1B, experimental spot size distributions from typical recall responses are shown for CD8 and CD4 cells producing the five cytokines tested. In each case, uni-modal bell-shaped curves were observed, consistent with a Normally Distributed spot size distribution.

### 3.2. Spot Size Distributions for CD8 and CD4 Cells Follow Log Normal Function

In all cases studied, spot size distributions on a logarithmic scale closely resembled bell shaped Gaussian curves. To test whether these distributions are indeed Log Normal, we subjected them to stringent statistical normality tests. The Kolmogorov–Smirnov Test’s null hypothesis states that samples come from a normally distributed population. We used this “goodness of fit” test to calculate the probability of experimental distributions being Normal, with a test significance level α = 0.05. In order to illustrate this, the experimental Log size distributions are overlaid with the theoretical Normal function, indicated by the red lines, in Figure 1B. For each of these examples, the *p*-value for the Kolmogorov–Smirnov goodness of fit test is shown, representing the likelihood that the experimental distribution is Log Normal function. For each of these examples, the p values were equal to or larger than the test significance level (0.05), indicating acceptance of the null hypothesis demonstrating normality of spot size distributions on a logarithmic scale. 

**Figure 2:**
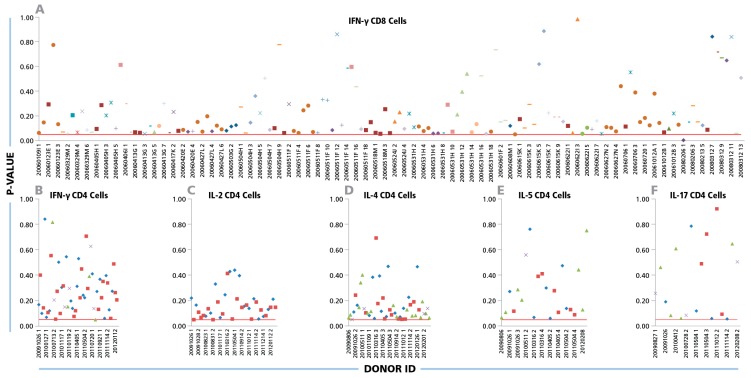
Log Normal size distributions for cytokine ELISPOTs produced by CD8 and CD4 cells. (**A**) For CD8 cells, IFN-γ ELISPOT size distributions were studied for a total of 334 positive recall responses induced by the 32 individual CEF peptides. Each peptide is represented by an individual symbol type. (**B**–**F**) For CD4 cells, 80 individual IFN-γ, IL-2, IL- 4, IL-5, and IL-17 spot size distributions elicited by inactivated CMV and EBV were studied; each one specified by a different symbol. The Kolmogorov–Smirnov goodness of fit test was used to determine the normality of spot size distribution for each individual positive recall response. Experimental IDs of the positive donors are shown on the x-axis, and the y-axis represents the p-values for the donor/peptide combinations. The red line indicates the cut-off significance level of 5%.

The Kolmogorov–Smirnov goodness of fit analysis was performed individually for all 334 positive CD8 cell IFN-γ recall responses. Figure 2A shows the p values obtained from each positive donor/peptide combination. With one exception (i.e. in 99.7% of the cases), the p-values were equal to or higher than the test significance level (α = 0.05). This is indicated in Figure 2A by the test cut off red line, thus the normality of size distributions on a logarithmic scale can be accepted. When CD4 cell recall responses measuring IFN-γ, IL-2, IL-4, IL-5, and IL-17 were subjected to the same analysis, Log Normal distributions were in 100% of test cases (Figure 2 B, C, D, and F, respectively). 

### 3.3. Spot Size Variation Among Donors, Antigens and Experiments

**Figure 3:**
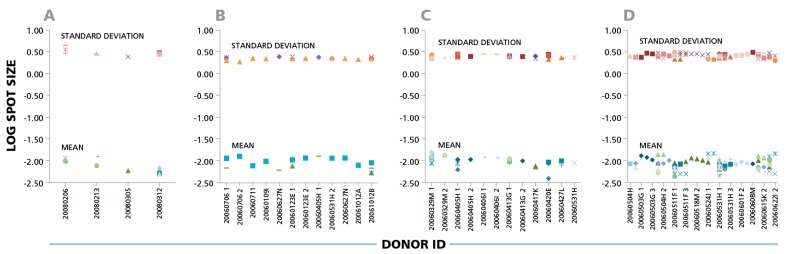
IFN-γ ELISPOTs produced by CD8 cells show a maximal three- to four-fold size variation between donors and antigens. Four independent experiments are shown in panels (**A**), (**B**), (**C**), and (**D**). IFN-**γ** CD8 responses of different donors against 32 individual CEF peptides are shown as different symbols. For each individual donor/peptide combination 1000 spots were analyzed. The mean spot sizes (mm^2^) and SD in the logarithmic scale (y- axis) are plotted *vs.* the PBMC donor’s ID (x-axis).

The data shown here suggest that parametric statistics can be used to establish spot size gates automatically for an individual donor/peptide combination. This can be of considerable practical relevance for batch counting, whether the same gates are applied for the analysis of one donor’s responses against different antigens, or for the responses of different donors to one antigen. Within individual experiments (four of which are shown in Figure 3), the mean spot size varied up to four-fold for different donors and peptides that elicited CD8 cell responses, corresponding to a 7.5% CV range on a logarithmic scale. Given that the spot sizes within a single distribution range from 100 to 300 fold (Figure 1), Normal Distribution suggests that spot size gates established for one donor/antigen combination can be applied to the counting of all results, when measuring the same cytokine. 

**Figure 4:**
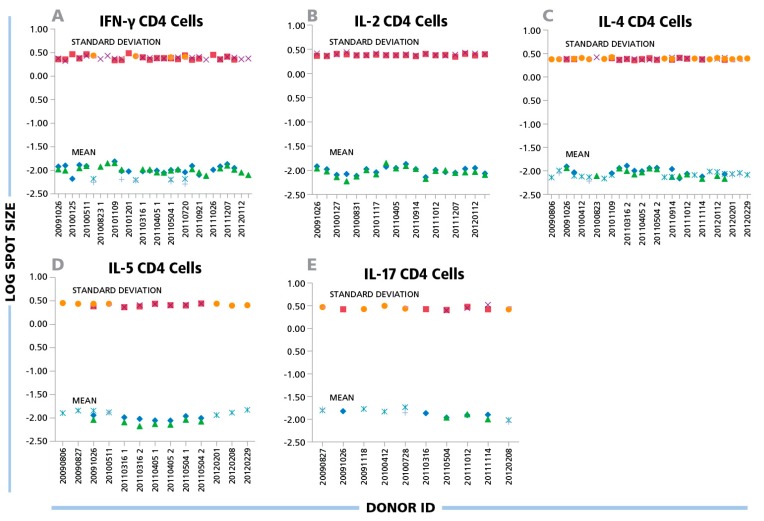
(**A**) IFN-γ, (**B**) IL-2, (**C**) IL-4, (**D**) IL-5, and (**E**) IL-17 ELISPOTs produced by CD4 cells show a maximal three-fold size variation between donors and antigens. Inactivated EBV or CMV virions (indicated by the different symbols) were used to induce CD4 cells of different donors to produce the cytokines specified in the panels. The mean spot size (mm^2^) and SD for each positive response (y-axis) are plotted in the logarithmic scale *vs.* the individual donors’ ID specified on the x-axis. For each data point 1000 spots were analyzed.

A similar variability was seen for protein antigens that triggered CD4 cells to secrete IFN-γ, IL-2, IL-4, IL-5 and IL-17: the mean spot size differences for CD4 responses were in the 6% CV range on the logarithmic scale for all cytokines tested (Figure 4). While the spot sizes for individual antigen/donor combinations were each Log Normally distributed thereby allowing the establishment of objective gating criteria for each, the gating criterion established for one donor or antigens will approximate, but will not perfectly match, other antigen/donor combinations.

**Figure 5:**
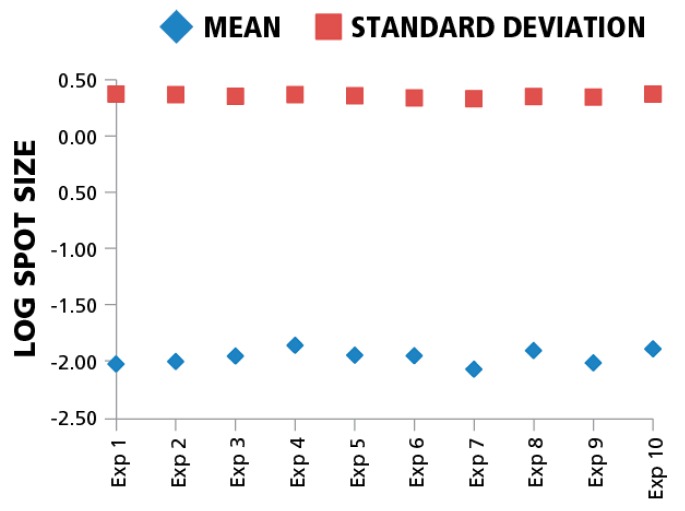
Mean spot sizes for the same donor/peptide combination show minimal variation between experiments. Ten independent experiments were performed testing repeatedly the HCMV pp65 induced IFN-γ recall response of the same PBMC donor. The mean spot sizes in mm^2^ (diamonds) and SD (squires) are shown in the logarithmic scale (y-axis) *vs.* experiment ID (x-axis). For each data point 1000 spots were used to build spot size distribution.

Since inter-assay variation, in particular the enzymatic-amplification reaction, can systematically affect spot sizes across different experiments, we also compared means and standard deviations of spot size distributions for the same donor/peptide combination from ten individual IFN-γ assays performed in the same laboratory at different time points. The variation of mean spot sizes and standard deviations of means (CV) between the assays in the logarithmic scale was approximately 4%. (Figure 5). Spot size distributions were reproduced reliably between experiments when standardized test conditions were used. 

### 3.4. Discussion

To our knowledge, this study represents the largest systematic analysis of T cell ELISPOT size distributions for recall responses in human PBMC. One hundred seventy two healthy subjects were tested for responses to a variety of recall antigens, resulting in a total of 334 CD8 and 80 CD4 test cases. For each of these individual test cases, stringent statistical analyses were applied to assess the probability that the observed spot size distributions fit Log Normal function. We found that the experimental spot size distributions of the CD8 cell as well as of the CD4 cell recall responses closely followed Log Normal distribution.

Accurate recognition of the individual spots is essential for the spot size analysis reported in this study. Spots produced by cytokine secreting cells fade from the center to the periphery and do not have defined edges reflecting the diffusion of the cytokine from the secreting cell. In order to define the size of spots unambiguously, therefore, the software needs to use a cutoff density value over the well background. Accurate assessment of spot sizes critically depends on the precise recognition of individual spots over the respective background coloration of the membrane, which often shows local variations throughout the membrane surface including a typical increase of background along the edge of the well. Thus, the counting software has to be able to perform accurate local dynamic background corrections. Another critical issue is a proper separation of closely situated overlapping spots. A failure to do so will result in spot clusters counted as individual spots, or if overdone, in cutting individual single spots into smaller fragment (that is, an over- or underestimation of the real spot sizes). Similarly, spot size distributions will be distorted if the software does not discern true spots (fading from the center to the periphery) based on their morphology from smaller artifacts (usually having sharp edges). The Log Normal size distribution-based automatic gating therefore can only be done with an ELISPOT reader that has the single cell-verified spot count- and analysis capabilities mentioned above. Whether or not a reader has that capability can be relatively easily verified by testing PBMC plated in serial dilutions. Accurate spot recognition occurs if the spot counts and the PBMC numbers plated follow a linear function in the 100,000 to 800,000 PBMC/well range (the range in which the PBMC form a monolayer on the membrane [16]), and if this linearity applies for high numbers of spots as well, that is, if spots are correctly recognized even when spots become confluent and the membrane background staining is elevated due to the ELISA effect. Such has been verified for the ImmunoSpot® system [11,16,17,18].

Most of the antigen induced cytokine responses in mixed cell populations (like PBMC), but not all, are known to include non-T cell derived undersized background spots and oversized spots resulting from T-cell clusters [12,17], constituting the very reason for size gating (in this study we analyzed all spots including these irrelevant categories on the tails of the size distributions). Therefore, inherently spot size distributions are “contaminated” on the tails and the selection of a proper statistics has to account for this. The Kolmogorov–Smirnov goodness of fit test is ideally suited to accommodate this situation because of its somewhat lower sensitivity on the tails of the distribution and good sensitivity in the middle part. In contrast, using methods sensitive to minor deviation from the normality on the tails of the distribution would result in low power detecting underlying Normal distributions of the relevant T cell derived spots. Indeed the p values that we obtained in the Kolmogorov–Smirnov goodness of fit tests were shifted toward smaller values (between 0.05 and 0.4, Figure 2), suggesting a minor but systematic deviation of the experimental distributions from perfectly symmetric Gaussian. This results from some of the analyzed distributions being slightly negatively skewed, with somewhat longer tails on the left side. This deviation, however, is negligible for the scope of this study whose purpose was to establish whether spot sizes follow a known distribution function closely enough to permit automated, statistics-based size gating. Spot size distributions that closely approximate Log Normal were found to be the rule for T cell ELISPOT results, providing an experimentally verified basis for statistics-based, objective ELISPOT counting.

The notion about Log Normal size distributions of ELISPOTs first surfaced when we studied IFN-γ spot formation by T cell clones in detail. When spots were counted using gating based on Log Normal distribution, the numbers of cloned T cells plated and the numbers of spots counted matched precisely, indicating that every single T cell was detected [5]. Therefore, in an experimental setting where the number of cytokine secreting T cells per well was known the accurate counting results validated the use of the Log Normal-based gating assumption. 

When spot sizes (in log scale) for a given T cell population are normally distributed, one can define spots belonging to a particular distribution with known probability. Spots that are outside the ± 3 SD interval of the mean spot size are with 99.73% likelihood not members of the same population, *i.e.*, are not produced by individual T cells. Larger spots (above + 3 SD) will result from cell clustering, and should not be counted as single events. Instead, an estimated count should be used, based on the number of average-sized spots that would be required to form a spot of that particular size. In many cases clustering can be avoided by using fewer cell per well. The number of oversized spots produced by individual T cells will linearly decrease with the numbers of cells plated (together with total spot counts), whereas oversized spots resulting from cell clusters, will be reduced much faster as the cells are diluted (for doublets, the relation is proportional to the square of cell numbers, for larger clusters, the function is more complex, and also drops more steeply). These rules hold for cell clusters randomly formed on the well bottom. If cell clustering has already occurred in the cell suspension prior to plating (e.g., as a result of free DNA causing clumping of cells), the proportion of clusters cannot be reduced by cell dilution and proper gating is the only option. The use of DNAse will experimentally eliminate such clusters. 

Spots that are smaller than −3 SD of the mean spot size are usually caused by non-T cell bystander cytokine secretion or by background staining artifacts, and should be gated out (ignored). Therefore, when spot sizes show Log Normal distribution, as was shown here, one can use an objective statistical approach to set gates for counting. This statistics-based approach forms the basis for the AutoGate™ function of the ImmunoSpot® Software [12,17]. 

Gating is not the only criterion required for objective ELISPOT counting. As can be seen in Figure 1A, the background coloration of the membrane in ELISPOT wells will differ for different experimental conditions dependent on the number of cytokine-producing cells per well, and on the net amount of cytokine they produce. Cytokine that is not captured directly around the secreting cells diffuses into the bulk solution, and is re-captured randomly on the membrane much as in an ELISA. Background coloration due to this “ELISA effect” can evenly affect the entire well bottom, but frequently it will be more pronounced in parts of the well where clusters of cytokine-secreting cells occur, and at the rim of wells. Without background corrections, faint spots in wells containing few cytokine-secreting cells can be less optically dense than the background in wells containing higher numbers of cytokine secreting cells. Therefore, counting of faint spots that occur over different levels of background coloration will not be possible with fixed intensity based sensitivity (thresholding). A well-designed ELISPOT counting software should thus be capable of correcting local background variations and also making well-to-well sensitivity adjustments. The ImmunoSpot® Software does both automatically with its BackgroundBalance™ and SmartWell™ features.

As a follow up to this study, we tested whether different investigators would come up with similar spot counts when analyzing the same ELISPOT plate using the Autogate™ function based on Log Normal size distributions [18]. The well image files of the plate were sent to nine participating laboratories. The plate contained wells with a wide range of spot numbers from the same antigen/donor combination, over various levels of background coloration. With the BackgroundBalance™ and SmartWell™ features activated, the investigators were permitted to select any of the 96 wells (containing any numbers of spots and background coloration) to present the ImmunoSpot® Software the “representative positive wells” for gating. The AutoGate™ function builds the experimental spot size distribution curve from these cumulative data, tests it for normality, and if normality is accepted, automatically sets the upper and lower gates. When the nine different investigators counted the same plate in this way, the spot counts for each of the 96 wells of the plate were within 7% (CV) of each other [18]. Therefore, using scientifically validated counting parameters established by the software, count variability among laboratories were reduced substantially compared to subjective (manual) parameters setup. 

While the individual antigen/donor combinations resulted in Log Normal distributions, there were differences in the mean values of the distributions between antigens and donors. According to the data in Figure 3, Figure 4 and Figure 5, the maximal difference between mean spot sizes was about three- to four-fold, which is much smaller than 100–300 fold difference within a single experimental distribution (Figure 1B). The CV of mean log spot sizes for different donor/antigen combination in the same experiment was between 4% and 7% (Figure 3 and Figure 4), Therefore, in the test cases we studied, gates set automatically by sampling a few different donor/peptide combinations could be used with considerable accuracy for most of the samples in the same experiment. It should be noted, however, that these results were obtained from testing healthy donors whose T cell activation *in vivo* occurred in the indefinite past, *i.e.*, the spot distributions studied were generated by resting memory cells. 

T cells that had recently been engaged in an immune response *in vivo*, e.g., due to recent infection or vaccination, produce more cytokine per cell than long term memory cells that have encountered antigen in the distant past resulting in Log Normal distributions that are shifted toward bigger spot sizes [14]. Accordingly, different size gates are needed to count T cells that are in the effector or memory state. When T cells are stimulated with a high dose of antigen (relative to the T cell’s functional affinity for antigen) they will produce more cytokine on a per cell basis than the same T cells stimulated with a low dose of antigen. However, in both cases the spot sizes are Log Normally distributed. [5]. Subsequently, for the same antigen dose, high affinity T cells can be expected to produce more cytokine on a per cell basis than low affinity T cells, and different gates might need to be used to count them correctly. Any spot size distribution that significantly differs from the “representative wells” that were selected for auto-gating must be set individually. This can be readily accomplished in the ImmunoSpot® Software using Multi-parameter Mode™ and verified/corrected for deviant responses in the Quality Control Mode™. 

The variability of mean spot sizes can also provide additional information that can be extracted from ELISPOT results. Typically, spot counts are the only parameter recorded, establishing the frequencies of cytokine secreting cells. Cytokine productivity is not commonly recorded but, as stated above, can provide important additional information on the T cell response, including its functional avidity and antigen-encounter history. Since the spot sizes follow Log Normal distribution in all of these instances, recording the mean spot size and the SD of an experimental distribution should suffice to characterize T cells’ secretory productivity, which we believe should become part of routine, automated ELISPOT data analysis. 

For protein antigens and short peptides uni-modal bell-shaped spot size curves were seen resulting from either CD4 or CD8 cells becoming activated. In more complex situations, when both cell types could potentially be activated at the same time (e.g. mitogen stimulation), experimental spot size distributions could be bi-modal or even multi-modal (e.g. when NK cells are stimulated along with CD4 and CD8 cells after PHA exposure). Although cumulative distributions for such mixed responses may deviate from Log Normal function, knowing that each of the components is normally distributed still permits statistical data analysis. So far, however, we observed only a few exceptions when spot sizes did not fit a uni-modal distribution. The ImmunoSpot^®^ Software Version 5.3, with the built in feature in the Quality Control module, can be used to verify whether gates set using sampled representative wells are applicable for all counted wells. If required, gating correction for deviant donor/antigen combinations can be performed automatically or overridden manually.

## 4. Conclusions 

The data establish that spot size distributions in T cell ELISPOT assays follow Log Normal distribution function for antigen-stimulated CD8 and CD4 cells secreting IFN-γ, IL-2, IL-4, IL-5 and IL-17. Therefore, size gates for counting can be set automatically, by means of statistical analysis, allowing for objective spot counts, as well as the harmonization of experimental results among different investigators.

## Abbreviations

IL: Interleukin;
IFN-γ: Interferon- gamma;
CMV: Cytomegalovirus;
EBV: Epstein-Barr virus;
PBMC: Peripheral Blood Mononuclear Cells;
SFU: Spot Forming Unit
